# Advances and Prospects in the Diagnosis and Treatment of Blood Culture-Negative Infective Endocarditis

**DOI:** 10.31083/RCM39211

**Published:** 2025-08-26

**Authors:** Wei Ji, Zhihuang Zhao, Xinjun Lin, Wanjun Huang, Qidong Chen, Yuwei Chen, Liyong Shi, Chaoxiang Xu, Xiaoyang Chen

**Affiliations:** ^1^The Second Clinical College of Fujian Medical University, 362000 Quanzhou, Fujian, China; ^2^Department of Pulmonary and Critical Care Medicine, Fujian Key Laboratory of Lung Stem Cell, The Second Affiliated Hospital of Fujian Medical University, 362000 Quanzhou, Fujian, China; ^3^Department of Cardiology, The Second Affiliated Hospital of Fujian Medical University, 362000 Quanzhou, Fujian, China

**Keywords:** BCNE, endocarditis, etiology, diagnose, treatment

## Abstract

Blood culture-negative infective endocarditis (BCNE) constitutes an important subtype of infective endocarditis. Despite the rarity of BCNE, this subtype poses a significant diagnostic challenge and promotes a high mortality rate. Recent advances in diagnostic modalities have facilitated the rapid identification of BCNE. Moreover, empiric diagnostic and therapeutic approaches, supported by intensive and rigorous epidemiological and observational investigations, have yielded positive results. There is a growing inclination in clinical management toward early surgical interventions while rigorously assessing surgical risks, complications, and anticipated benefits. This review examines the epidemiology, microbiological data, and diagnoses of medical and surgical BCNE in contemporary practices.

## 1. Introduction

Infective endocarditis (IE) is a serious global public health concern [1]. Despite the low incidence (affecting approximately 1–10 individuals per 100,000 annually worldwide), the associated hospital mortality can be as high as 40% [2]. The prevalence of IE continues to rise, with cases increasing from 400,000 in 1990, to more than 1 million in 2019 [3]. IE is characterized by the infection of the endocardium by pathogenic microorganisms. IE pathogenesis requires three key elements: susceptible anatomical substrates for bacterial colonization, entry of pathogens via the bloodstream, and compromised host immunity [1]. The disease progresses through four sequential phases [1, 4, 5]: (1) Endothelial injury exposing subendothelial matrix proteins, forming sterile thrombi that serve as microbial adhesion sites; (2) Transient bacteremia enabling pathogens to attach to the damaged endothelium; (3) Microbial surface adhesin-mediated binding to host extracellular matrix components; (4) Mature biofilm development on valvular surfaces, resulting in immune evasion and antimicrobial resistance.

**Fig. 1.**
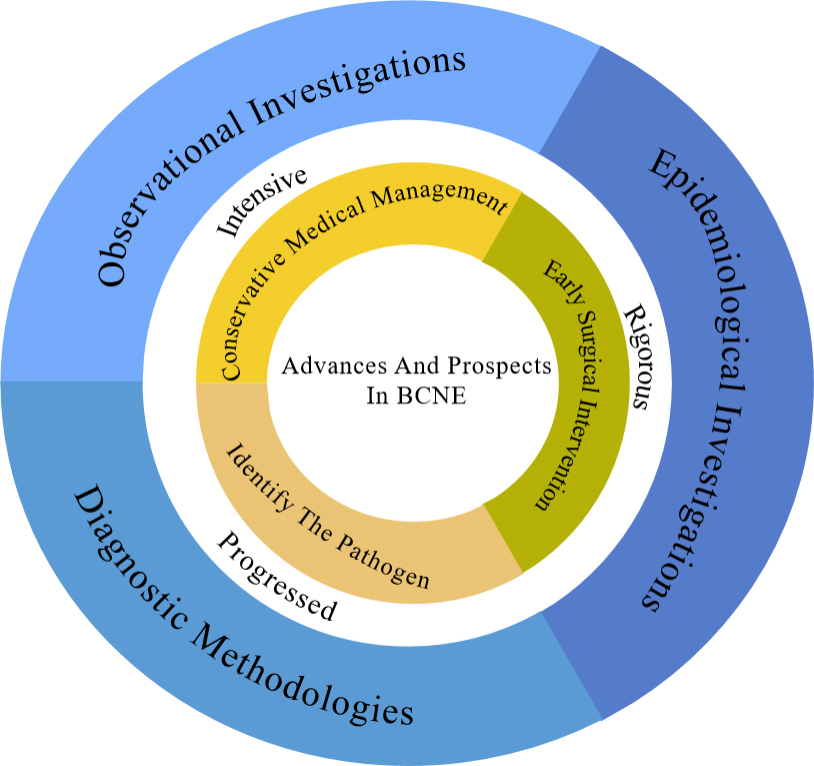
**The figure illustrates that this review discusses the evolving epidemiology, observational studies, and diagnostic methods in the BCNE, and describes the current status of its empirical diagnosis and treatment versus standardized diagnosis with changing etiology, with a preliminary description of its progress and promise**. BCNE, blood culture-negative infective endocarditis.

Amongst cases of IE, there is the distinct entity known as blood culture-negative infective endocarditis (BCNE), where traditional blood culture methods fail to isolate the causative pathogens [6, 7]. BCNE presents clinicians with a significant diagnostic and therapeutic challenge and is associated with a higher mortality rate than blood culture-positive infective endocarditis (BCPE) in patients managed medically (excluding those undergoing surgical intervention) [8]. The diagnosis of BCNE is challenging and contributes to increased mortality, since it may delay the initiation of therapeutic interventions [4, 7, 8]. Nevertheless, recent advances in diagnostic modalities, improved access to valve tissue, and insights gleaned from epidemiological studies hold promise for the early diagnosis and prompt management of BCNE [9] (Fig. 1).

## 2. Epidemiology and Definition

Due to the considerable challenges in the diagnosis of BCNE, an accurate estimation of its incidence remains a daunting task. Since its initial description in the 16th century, the epidemiological characteristics of infective endocarditis have undergone profound transformation. This has been influenced by the ongoing development of antibiotic resistance, the identification of new at-risk populations, and advancements in medical practice [10, 11]. Notably, males exhibit a two-fold increase in disease susceptibility compared to females [12, 13]. Currently, BCNE accounts for 10–20% of all IE cases [11, 14, 15]. However, in certain regions, antibiotic prescribing practices have resulted in BCNE cases exceeding that of BCPE. In a study conducted in northeastern Thailand from 2010 to 2012, BCNE accounted for 54.5% of the total IE cases [16]. A South Korean investigation (2016–2020) identified 40 BCNE cases (24.5%) among 163 patients with IE or large vessel vasculitis [17]. Concurrently, South African research (2019–2020) reported BCNE in 14 of 44 patients (31.8%) with confirmed or suspected IE [18]. Nationwide Danish registries (2010–2017) documented BCNE in 778 of 4123 IE cases (18.9%) [19], while a Spanish study (1984–2018) observed a significantly lower prevalence, with 83 BCNE cases (8.3%) among 1001 IE patients [20].

BCNE is divided into three categories [7, 9]. First, bacterial endocarditis, typically caused by streptococci, enterococci and staphylococci, where the absence of microorganisms in culture is often attributed to the initiation of antibiotic treatment prior to testing. Second endocarditis related to fastidious organisms, commonly associated with the HACEK-like bacteria (*Haemophilus*, *Aggregatibacter*, *Cardiobacterium*, *Eikenella*, *Kingella*), *Mycobacterium*, nutritionally variant streptococci, fungi (*Candida spp*) and some eukaryotic infections (*Echinococcus granulosus*). Identification of these organisms often requires an extended period of culture spanning several days. Third, the “true” blood culture-negative endocarditis: The etiological agents of this category are typically intracellular bacteria that cannot be cultured from blood samples. Common causative agents include *Bartonella*, *Coxiella burnetii*, and *Tropheryma whipplei*. *Bartonella* and *Coxiella* can be diagnosed with specific serological testing, and *Tropheryma* can be identified via Polymerase Chain Reaction (PCR) of infective heart valve tissue obtained during surgery.

## 3. Microbiology

The predominant pathogens previously linked to BCNE were the HACEK-like bacteria, nutritionally variant streptococci and *Streptococcus anisopliae*. However, contemporary analysis has indicated a shift towards pathogens such as *Coxiella burnetii*, *Bartonella*, and fungi [15]. Although *streptococci* and *staphylococci spp* are key etiological agents in BCPE, they are also significantly contributing to the pathogenesis of BCNE and should not be underestimated [9]. Furthermore, certain rare yet highly lethal pathogens, such as *Mycobacterium*, warrant serious consideration (Table 1).

**Table 1.** **Partial clinical features of different pathogens of BCNE**.

Pathogen	Possible heat patterns	Vulnerable heart valves	Partial risk factors
HACEK-Like Bacteria	Can be intermittent fever	Natural valves (mitral, aortic)	History of oral infection, Dental manipulation, Periodontal disease
Non-Tuberculous	Can be low or no fever	Prosthetic valve/right heart system	Immunosuppression, Hemodialysis, Central venous catheter, History of cardiac surgery
Tropheryma Whipplei	Mostly without fever	Aortic valve	History of gastrointestinal disorders, Malnutrition
*Bartonella*	Can be low or no fever	Left heart valve (aortic valve predominant)	Stray animal exposure, Immunosuppression
Q fever	Chronic hypothermia	Aortic valve	Livestock/Pet contact, Agricultural area residence
Aspergillus	Can have high fever	Prosthetic valve/right heart system	History of cardiac surgery, Immunosuppression, Long-term broad-spectrum antibiotic use
Candida	Can have fever	Prosthetic valve/right heart system	Prolonged intravenous catheterization, Immunosuppression, Broad-spectrum antibiotic use

Marantic endocarditis (ME) warrants particular attention. This nonbacterial thrombotic endocarditis manifests as sterile valvular vegetations unassociated with bacteremia, however they exhibit several clinical features associated with IE [21]. Shared features include fever, cardiac murmurs, and valvular masses seen on echocardiography [21, 22]. ME should be considered when patients present with this triad along with negative blood cultures. Malignancy—particularly mucinous adenocarcinomas or lymphomas—are the predominant risk factor, which differentiates it from patients with IE [21].

### 3.1 Bacterial BCNE

HACEK-like bacteria are commensal organisms found in the human oral cavity and upper respiratory tract, typically exhibiting low pathogenicity [12]. This group comprises *Haemophilus parainfluenzae*, *Aggregatibacter spp*, *Cardiobacterium hominis and valvarum*, *Eikenella corrodens*, *Kingella kingae*, and *denitrificans* [23]. These microorganisms, known for their fastidious nature, require nutrient-rich media for culture and are characterized by slow growth. Previously, the suspicion of BCNE related to these organisms required a culture period of two weeks. Recent studies indicate that the average isolation time for these pathogens is less than 5 days, diminishing the benefits of such prolonged incubation times [24, 25, 26, 27]. The mechanism by which these pathogens cause endocarditis is that after colonizing the oral cavity or upper respiratory tract, they access the vascular space during events such as dental procedures, localized infections, trauma or in the context of chronic periodontal disease [28, 29, 30]. In contrast to BCNE, HACEK-like pathogens can cause conditions such as otitis media, oral infections and bacteremia [31, 32]. Notably, due to their relatively low virulence, endocarditis resulting from these organisms typically carries a favorable prognosis, whether it affects native or prosthetic valves [12, 33].

Endocarditis due to *Mycobacterium spp,* is uncommon [34]. The Mycobacteria responsible for IE typically belong to the fast-growing, non-tuberculous group (e.g., Mycobacterium chelonae and Mycobacterium abscessus) [35]. Common risk factors for such infections include: the presence of indwelling hemodialysis catheters, central venous access devices, immunocompromised states, immunosuppressive medications, administration of TNF-a antagonists, and arthroplasty procedures [35, 36]. Mycobacterium avium-associated BCNE typically manifests with non-specific clinical features. Patients commonly present with dyspnea and low-grade fever, though fever patterns often dissociate from disease progression. Cardiac auscultation may reveal murmurs in select cases [34]. In cases of BCNE linked to Mycobacterium, there is often extensive antibiotic resistance, which, when coupled with the challenges associated with identification, leads to delays in diagnosis and treatment, and may contribute to the significantly higher mortality rate [35]. Early surgical intervention in this form of BCNE is believed to result in decreased mortality [37].

*Tropheryma whipplei* is another pathogen associated with BCNE [38]. Initially discovered in 1907, *Tropheryma whipplei* was not classified as a Gram-positive bacterium until the late 20th century [39]. BCNE resulting from *Tropheryma whipplei* tends to progress more slowly, resembling that of BCNE due to *Bartonella* and Q fever [40]. It is important to note that the clinical presentation of this form of BCNE is often non-specific, although typical signs of Whipple disease may be observed (gastrointestinal disturbances, weight loss, joint pain and central nervous system changes) [39, 41, 42]. Individuals with this form of BCNE often exhibit a predisposition toward chronic inflammation, and may present without fever and with low inflammatory markers [38, 43]. The diagnostic complexity of *Tropheryma whipplei* BCNE is compounded by the reliance on PCR tissue samples for diagnostic confirmation. Due to its rarity and the diagnostic challenges, treatment regimens are often based on limited case reports or observational reports rather than randomized clinical trials [38]. The prognosis for this variant of BCNE is typically poor.

*Bartonella spp* is a common cause of BCNE. It is a Gram-negative, intracellular organism [7, 44]. There are more than 30 known species of Bartonella, at least eight of which can cause BCNE. There is significant geographical variation, with species in Ecuador and Peru causing particularly life-threatening disease [44, 45, 46, 47]. The clinical presentation of *bartonella* infection is non-specific, and infection is often diagnosed at the onset of cardiogenic shock. The mortality rate for this form of BCNE is high, ranging from 7–30%, which may be due to the progression of the disease at time of diagnosis [18, 48]. *Bartonella* BCNE often requires surgical management in addition to standard antimicrobial therapy [44].

### 3.2 Fungal BCNE

Fungal endocarditis accounts for less than 3% of infective endocarditis. *Candida* and *Aspergillus* are the most common pathogens in this group [49]. *Candida* fungemia typically yields positive blood cultures, and *Aspergillus* is the primary fungal BCNE. *Candida*endocarditis lacks classic symptomatology, with <70% of patients exhibiting fever. Fatigue, weight loss, and chills have a higher prevalence compared to bacterial endocarditis [49]. *Aspergillus*-associated fungal endocarditis presents with fever, cardiac decompensation, dyspnea, murmurs, and peripheral embolization—particularly neurological deficits from septic emboli [50]. Positive *Aspergillus* cultures often reflect contamination of culture medium rather than true infection [51]. Cardiac surgery is a major independent risk factor for *Aspergillus* BCNE [52, 53]. Diagnosis of *Aspergillus* BCNE poses a significant challenge, and is usually delayed. Studies have shown that over a third of these cases (sample size 240) are diagnosed posthumously [49, 53]. Definitive diagnoses requires excised valve tissue or the presence of mycotic emboli [49]. Successful treatment requires both medical and surgical intervention. In a study involving 53 cases, only 2 individuals (4%) survived with antifungal treatment alone, while 17 patients (32%) survived through a combination of medical and surgical approaches [54]. Aspergillus-associated BCNE carries both a high mortality and propensity for recurrence, necessitating lifelong treatment [49, 53].

*Malassezia*, an opportunistic pathogen, is a fungus typically present in normal skin flora [55]. BCNE stemming from *Malassezia* is rare and is associated with an atypical clinical presentation. A study of three cases of *Malassezia* BCNE found the primary symptoms to be heart failure and fever [56]. The prognosis in these cases was poor.

### 3.3 Atypical Pathogen-associated BCNE

*Coxiella burnetii* is an intracellular organism which is a common culprit of BCNE [57]. The clinical presentation is usually related to a chronic Q fever; thus, it is commonly referred to as “Q fever-associated BCNE” [58]. Though more than half of the affected individuals exhibit heart failure and fever, there are few specific clinical findings [59]. The presence of a fever differentiates *Coxiella* BCNE from that of *Bartonella* and *Tropheryma whipplei*, the other causes of chronic BCNE. Similar other causes of BCNE, delayed diagnosis may contribute to a poor prognosis [57, 60]. Diagnostic delay is attributed to the challenge of serological testing—serum antibodies may not be detectable until 1–2 weeks post-infection [61]. Echocardiography has limited efficacy in this form of BCNE, with abnormal echocardiographic findings reported in only 12% of cases [57, 60]. PCR diagnostic methods have been disappointing, with suboptimal sensitivity [58]. A study of 100 cases showed positive PCR results in only 18% of patients [62]. Lifelong antibiotic therapy is recommended for the treatment of Q fever BCNE. Some studies have advocated for surgical intervention, as medical therapy does not guarantee complete resolution [57, 63].

*Mycoplasma* BCNE is exceedingly uncommon [64]. Mycoplasmas are pathogenic microorganisms inhabiting the human genitourinary tract, and are a diagnostic challenge since culturing them is difficult and their structure is atypical [40, 65]. Prompt adjustment of antibiotic therapy upon identification averts fatal outcomes for patients with *Mycoplasma* BCNE [64, 66]. BCNE linked to *Legionella* and *Chlamydia* has also been reported, but is rare [67, 68].

## 4. Diagnosis

Two decades have passed since the inception of the original Duke criteria for the diagnosis of IE [69]. As the manifestations and presentation of IE change over time, so have the Duke criteria. The International Society for Cardiovascular Infectious Diseases (ISCVID) revised the criteria in 2023 [70]. These changes have been reflected in the most recent European Society of Cardiology (ESC) guidelines [1]. Both the modified Duke criteria and the ESC guidelines offer a diagnostic framework founded on clinical presentation, microbiological insights, and imaging modalities.

**Fig. 2.**
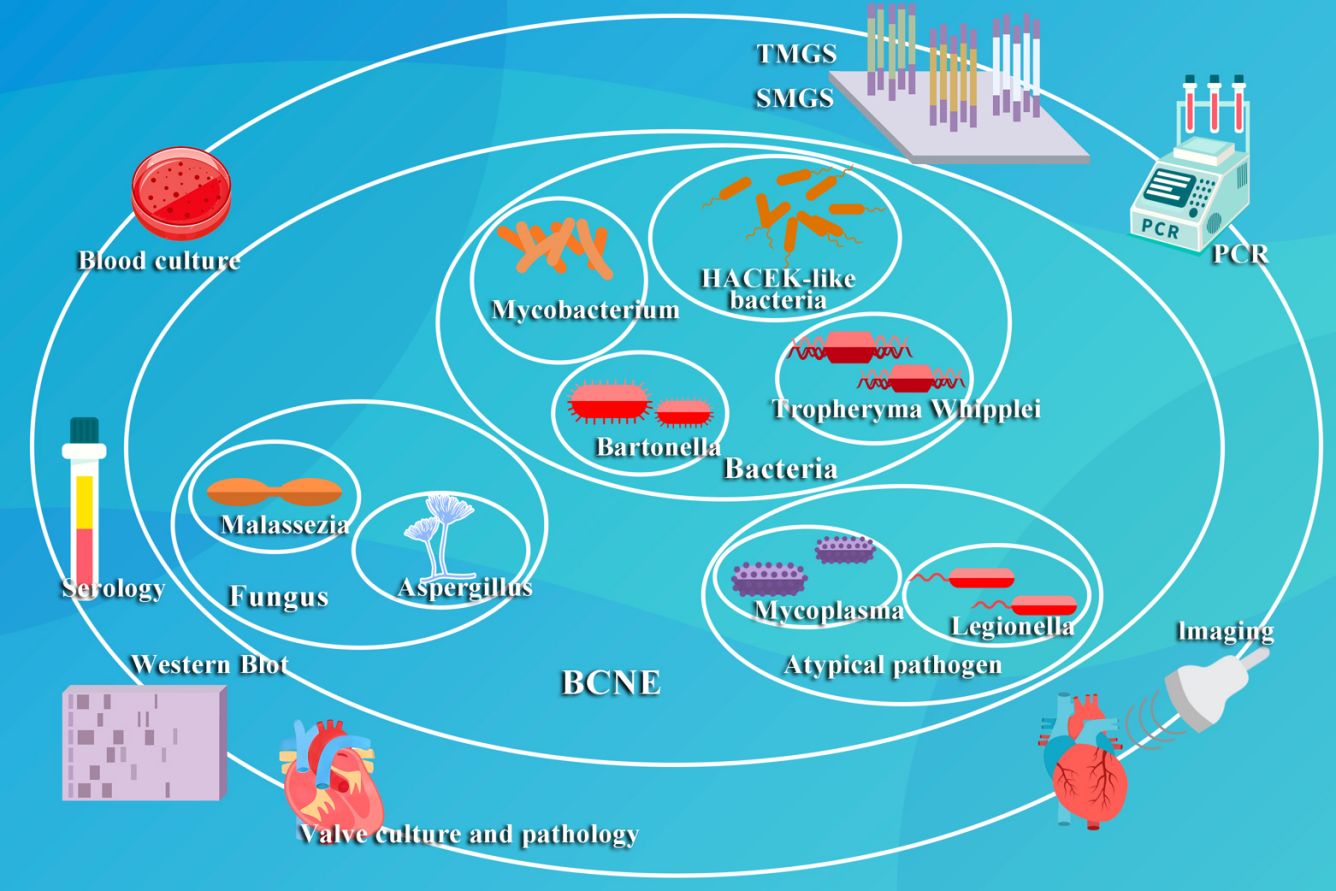
**This figure illustrates the prevalent pathogenetic classifications and established diagnostic methods for BCNE**. TMGS, targeted metagenomics sequencing; SMGS, shotgun metagenomics sequencing; PCR, Polymerase Chain Reaction.

Although blood cultures have traditionally served as the gold standard for the diagnosis of IE, the difficulties associated with culturing organisms in BCNE have prompted the exploration of alternative diagnostic approaches. Although there is a risk of false positives with serological assays, they play a crucial role in identifying pathogens such as *Coxiella burnetii* and *Bartonella*. Imaging techniques, notably echocardiography, are critical when blood cultures have yielded no growth. 18F-FDG PET has emerged as an important diagnostic tool when echocardiography and cultures are inconclusive, yet the clinical suspicion is high. Tissue culture and histopathology of heart valves offer valuable insights to support the diagnostic process. Molecular diagnostic methods, with their increased sensitivity, specificity and rapidity, are quickly emerging as valuable technologies. The use of multiple modalities may represent the most effective strategy, with reports of a 97% identification rate when targeted metagenomics sequencing (TMGS), shotgun metagenomics sequencing (SMGS) and blood culture techniques are combined [71]. The field of pathogen diagnostics in BCNE appears encouraging, heralding a future marked by enhanced diagnostic precision and efficacy and the potential for limiting the costs for these diagnostic techniques (Fig. 2).

### 4.1 Blood Culture

IE can be stratified into BCPE and BCNE, based on the results of blood cultures. To obtain accurate results, it is recommended that multiple sets of blood cultures are drawn from peripheral sites prior to the administration of antibiotics. Blood should be cultured in both aerobic and anaerobic environments, and examined with Gram stains [16, 72]. Even a single positive blood culture should be approached with caution. While the identification of typical organisms can typically be achieved within two days, fastidious or atypical pathogens may require a longer incubation period. For example, identification of the *Cutibacterium* species requires an extended culture up to 14 days, even if there is no growth after 5 days [73]. Such prolonged incubation periods can result in treatment delays.

### 4.2 Serology

Serological tests should be ordered 48-hours after a negative blood culture. Since *Coxiella burnetii* and *Bartonella* are the predominant pathogens associated with BCNE, it is important to consider these pathogens where the IgG phase I titer of the pathogen exceeds >1:8000 [1, 74]. In regions with a high prevalence of *brucellosis*, serological testing is strongly recommended [7]. Not all pathogenic organisms warrant serological testing; pathogens such as *Chlamydia, Chlamydophila* and *Legionella* often yield false positive results, particularly in cases where there is a history of previous infections.

### 4.3 Radiographic

Imaging plays a pivotal role in the diagnosis of BCNE. Echocardiography has emerged as a rapid and non-invasive screening modality. Transthoracic echocardiography (TTE) not only aids in the detection of the valvular abnormalities that support a diagnosis of BCNE, but also facilitates the identification of associated cardiac complications. When TTE results are inconclusive, but clinical suspicion is high, transesophageal echocardiography (TEE) may be warranted [75]. While TTE is suitable for all individuals with suspected BCNE, TEE is a more precise diagnostic modality [76, 77, 78]. It is important to acknowledge that a negative echocardiographic study does not definitively rule-out BCNE; false negatives can occur with elusive pathogens or small abscesses. In cases where clinical suspicion is high and echocardiography is negative, 18F-FDG PET-CT imaging can be a valuable adjunct, demonstrating a diagnostic sensitivity of 91–97% [79, 80]. However, imaging modalities can only evaluate the effectiveness of antibiotic treatment through the observation of changes in vegetations, and can be difficult to use to guide medical management [81]. Imaging plays a critical role in detecting systemic complications of IE, including emboli and metastatic abscesses. The EURO-ENDO registry demonstrated embolic events occur in 40% of IE cases, correlating with elevated morbidity and mortality [82]. Whole-body CT imaging identified extracardiac lesions in 798 of 1656 IE patients (48.2%) [83], underscoring the value of comprehensive imaging protocols for assessing systemic involvement and localizing sources of occult infections [3].

### 4.4 Tissue Culture and Histopathology of Heart Valves

It is recommended that patients who undergo cardiac surgery undergo post-surgical microbiological and histopathological assessment of heart valve tissue. Even though the tissue culture has a low sensitivity (6–26%), it remains an important diagnostic tool for the identification of BCNE [84]. Histopathologic studies typically employ haematoxylin and eosin stains, which aid in the recognition of inflammatory responses, offer insights into specific microorganisms, and assist in the diagnosis of non-infectious etiologies. Specialized histological stains, such as the Warthin-Starry silver stain for *Bartonella*, periodic acid-Schiff for *Tropheryma whipplei*, or methenamine silver for fungal identification, can be employed to pinpoint specific pathogens [40].

Immunohistochemistry has proved invaluable for identifying *Bartonella* and *Chlamydia* within valve tissue, utilizing capture enzyme-linked immunosorbent/immunofluorescence assays (ELISA/ELIFA), immune-peroxidase staining, and direct immunofluorescence with fluorescein coupled monoclonal antibodies [85].

### 4.5 Molecular Diagnostics

Molecular methods present a highly sensitive and specific alternative to blood cultures in the diagnosis of BCNE, with the added benefit of a significantly faster turnaround. Molecular techniques encompass PCR assays targeting specific microorganisms, broad-spectrum PCR (focusing on 16S rRNA genes for bacteria and 18S rRNA genes for fungi), TMGS and SMGS.

#### 4.5.1 PCR Technology

The landscape of BCNE diagnosis has much promise, with significant cost reductions in newer, more sophisticated diagnostic modalities. PCR assays on valve tissue tend to exhibit greater sensitivity than that on blood specimens, as the concentration of microbial genetic tissue on valve specimens is greater. The *Bartonella* PCR assay demonstrates a sensitivity of 92% on heart valve tissue, but only 36% in blood and serum [86]. However, heart valve tissue is not available in all patients with BCNE, and thus testing of blood samples is often necessary. Organism-specific PCR primers target precise sequences of specific organisms, exclusively amplifying their DNA material. This targeted approach results in high specificity as it only identifies known microorganisms. Therefore, specific PCR assays designed for pathogens commonly associated with BCNE are invaluable for etiological diagnosis [74].

PCR techniques have demonstrated efficacy in the identification of many organisms, including *Tropheryma whipplei*, *Bartonella*, *Streptococcus*, *Streptococcus gallolyticus*, *Streptococcus salivarius*, *Streptococcus mutans*, *Enterococcus faecalis*, *Streptococcus sanguinis*, and *Staphylococcus aureus* [87, 88]. Numerous studies have shown the utility of broad-spectrum PCR technology in the diagnosis of BCNE. Boussier *et al*. [89] demonstrated that a two-step broad-spectrum PCR approach augmented BCNE diagnosis by over a third (37.5%) compared to heart valve cultures. Armstrong *et al*. [90] found that 41% of BCNE cases could be re-evaluated through 16S rDNA PCR, offering a more precise diagnosis of the pathogen. Rodríguez-García *et al*. [91] identified the causative agents in 36% of BCNE using the same methodology. Maneg *et al*. [92] revealed that broad-spectrum PCR conferred an additional 21.5% of BCNE diagnoses in patients with negative tissue or culture results. Marsch *et al*. [93] implemented PCR on heart valve samples in patients with negative blood cultures or inconclusive perioperative microbiological findings, which resulted in alterations in antibiotic regimens in 15.3% of cases. In a prospective study, broad-spectrum 16S rRNA gene PCR achieved 100% specificity and a 42.9% diagnosis for culture-negative bacterial infections [94]. It is important to note that chronic Q-fever associated prosthetic valve endocarditis (PVE) requires explanted valve tissue for 16s rRNA PCR identification [95]. PCR techniques are limited, however, due to their high susceptibility to contamination, false positives, and failure due to low DNA concentration in samples [96].

#### 4.5.2 TMGS and SMGS

The advent of Next-Generation Sequencing has propelled SMGS and TMGS into the forefront of BCNE diagnosis. These innovative methodologies offer a novel diagnostic approach for individuals unable to undergo cardiac surgery by detecting microbial cell-free DNA in plasma. The study indicates that SMGS surpasses the sensitivity of conventional 16S PCR—though performance is comparable to TMGS when detecting bacterial in whole blood and plasma [71]. TMGS can achieve a positive detection rate in 83% of BCNE patients, irrespective of prior antibiotic exposure [97].

The fundamental principle of microbial nucleic acid-based detection in TMGS and SMGS allows for detection which is independent of previous antibiotic treatment. However, these techniques are limited since they cannot distinguish between dead and live bacteria. *Streptococcus* and *Staphyloccocus aureus* are the most commonly identified pathogens with SMGS. SMGS has several advantages, including the ability to detect non-bacterial entities such as fungi, and can identify resistance genes and quantify bacterial DNA [10]. TMGS offers a cost-effective alternative.

#### 4.5.3 Western Blot

Western Blot (WB) is the gold-standard technique for quantifying protein expression. In a study by Arregle *et al*. [98], WB was employed to scrutinize the antigenic profiles of four prominent BCNE pathogens, leading to the identification of four potential pathogens in 14 unresolved BCNE cases. Their findings indicate that WB is an effective strategy for the diagnosis of BCNE associated with *Enterococcus faecalis* or *Streptococcus cholerae* [98].

## 5. Treatment

### 5.1 Medical Management

**Fig. 3.**
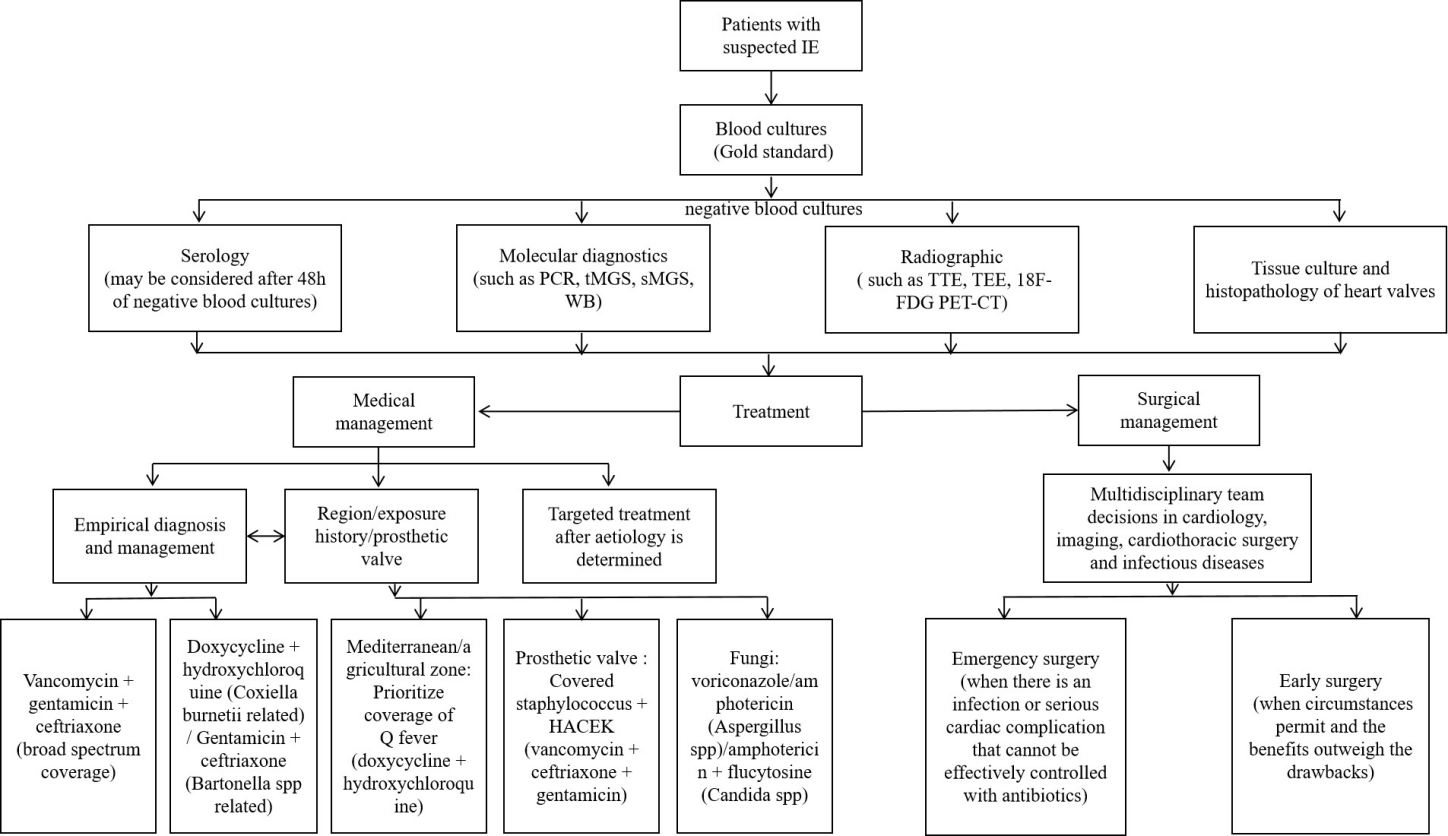
**BCNE diagnosis and treatment flow chart**. The chart contains the diagnostic and therapeutic processes that can be considered for patients with suspected IE when blood cultures are negative. IE, infective endocarditis; TTE, transthoracic echocardiography; TEE, transesophageal echocardiography; WB, Western Blot; 18F-FDG PET-CT, fluorine-18 fluorodeoxyglucose positron emission tomography – computed tomography.

The prognosis of BCNE is significantly improved by timely diagnosis and treatment, but the diagnosis is often delayed. Thus, in conjunction with improvements in diagnostic techniques, empiric treatment is vital [4]. Empiric treatment may encompass both medical and surgical interventions [8]. Even seasoned clinicians may have difficulty selecting the optimal therapeutic regimen for BCNE. Clinicians should carefully choose agents which offer a broad coverage of potential pathogens, guided by symptomology, epidemiology and a comprehensive medical history. Clinicians should also aim to minimize the use of nephrotoxins such as aminoglycosides [4]. The standard duration of intravenous antimicrobial therapy post-BCNE is typically 6 weeks, with prolonged treatment regimens often necessary for aggressive infections and refractory microbial strains (Fig. 3).

In the past, HACEK-like pathogens have generally been susceptible to ampicillin [4]. However, the increasing prevalence of β-lactamase-producing strains, and BCNE caused by HACEK-like pathogens, should be considered ampicillin-resistant until susceptibility has been confirmed [4, 99]. Nearly all strains of HACEK pathogens are sensitive to third and fourth-generation cephalosporins [100]. Fluroquinolones also exhibit coverage of these pathogens, though their use for the treatment of BCNE is limited to case studies. They can be considered as alternatives where cephalosporins are not susceptible to the infecting organisms [101]. Aminoglycosides are no longer recommended due to concerns regarding nephrotoxicity [4].

BCNE attributed to non-tuberculous mycobacteria frequently exhibits extensive drug resistance, and diagnosis is often delayed [34, 102]. Treatment for this condition is largely empirical, and empiric treatment with a multi-drug regimen should be instituted with adjustments made based on the results of anti-microbial susceptibility testing. Amikacin is often the most effective agent, with linezolid, imipenem and clarithromycin being feasible alternatives [37]. Standard anti-tuberculosis regimens (isoniazid, rifampicin, ethambutol, pyrazinamide) remain essential, with select patients requiring lifelong maintenance therapy [34]. In severe cases, surgical intervention may be necessary [37, 103].

BCNE associated with *Tropheryma whipplei* is often delayed until after heart valve replacement, and carries an extremely high mortality rate. Several studies have proposed a short-term regimen of penicillin or ceftriaxone, followed by ongoing administration of trimethoprim-sulfamethoxazole for at least a year, potentially alternating with doxycycline [38]. However, the optimal management strategy has not yet been established.

For Bartonella-associated BCNE, the American Heart Association (AHA) recommends the consideration of gentamicin and doxycycline when the diagnosis is confirmed [104]. In cases where Bartonella is suspected to be the sole pathogen, and given the effectiveness of ceftriaxone against other forms of BCNE, ceftriaxone may be utilized in lieu of doxycycline [105]. Bartonella predominantly affects patients with valvular disease, and more than 90% of these patients require surgical intervention along with medical therapy [105, 106].

In fungal endocarditis, amphotericin has historically been the preferred therapy [4]. However, a prospective randomized trial on Aspergillus BCNE showed that use of voriconazole was associated with a lower mortality (47.2% vs 68.4%) [107]. Therapeutic alternatives for *Aspergillus*-associated BCNE include itraconazole or posaconazole [49]. *Candida* endocarditis typically requires amphotericin B-based regimens [49]. Recent evidence indicates amphotericin B combined with flucytosine may enhance clinical outcomes, though without statistical significance versus monotherapy [108]. The use of echinocandins may also be considered [109], though they may not achieve optimal tissue concentrations within the central nervous system [110]. Antifungal therapy is typically continued beyond 6 weeks. Lifelong antifungal treatment is often recommended, given the substantial recurrence and mortality rates [111, 112].

A combination of tetracycline and fluroquinolones is the optimal treatment strategy for Q-fever BCNE [113, 114]. A tetracycline in combination with hydroxychloroquine is an alternative if fluroquinolones are contraindicated. Single-agent tetracycline or fluroquinolone therapy is not recommended. Triple therapy (tetracycline, fluroquinolone and hydroxychloroquine) has not demonstrated a significant advantage over standard therapy [113].

### 5.2 Surgical Management

The objective of surgery is excision of the infective tissue and repair or replacement of the affected heart valves [1]. If antibiotics prove ineffective at achieving control of the infection, or when serious cardiac complications arise, surgical management becomes imperative. Congestive heart failure, intracardiac abscess, atrioventricular block and fungal aneurysms may precipitate uncontrollable infections, requiring prompt surgical intervention [115]. Primary and recurrent embolic events are complications which require immediate attention. Vegetations exceeding 10 mm in size are predictive of embolic events, with an even higher risk in very large vegetations (>15 mm). These phenomena should prompt urgent surgical intervention [116].

In 20% to 40% of cases, IE is associated with a stroke [117]. In these cases, non-surgical management results in higher mortality [118]. The 2023 ESC guidelines recommend prompt surgical intervention where active infective endocarditis coincides with intracranial hemorrhage stemming from heart failure, transient ischemic events or stroke [99].

The optimal timing for surgical intervention remains unclear and warrants additional research [4]. The ESC and AHA have differing criteria on what constitutes early surgical intervention [119]. The AHA characterizes early surgery as an operation conducted during hospitalization but before completion of a course of antibiotics, while the ESC guidelines define surgery intervention as immediate (within 24 hours), subacute (within a few days), or elective (following at least 1–2 weeks of antibiotic therapy) [120]. Though there is no definitive recommendation, all guidelines underscore the need for a multidisciplinary approach, involving cardiology, radiologists, cardiothoracic surgeons and infectious disease specialists [99].

Current surgical indications and timing for infective endocarditis management are largely based on the 2023 ESC guidelines [1]. Perioperative risk stratification constitutes a critical determinant of therapeutic decisions, as clinically inoperable patients with guideline-defined surgical indications exhibit significantly poorer outcomes due to prohibitive operative risks [3]. There have been a number of studies investigating the effects of early surgical intervention. A series of observational studies conducted between 2007 and 2013 indicated that early surgical management improves mortality in patient with IE [121, 122, 123, 124]. A prospective randomized trial conducted in 2012 indicated decreased mortality in patients with IE who underwent early surgery compared to conservative treatment with antibiotics [119]. Anantha Narayanan *et al*. [125]conducted a meta-analysis of 21 observational studies and showed that early surgical intervention was associated with decreased mortality in IE. Root abscesses and valve dehiscence manifest in 60% of prosthetic valve endocarditis, and surgical intervention enhances long-term survival, mitigates recurrence, and decreases the need for repeated surgical intervention [126]. The ESC guidelines recommend surgical management for early PVE occurring ≤6 months post-implantation, entailing complete debridement and prosthetic valve replacement [1]. These outcomes may be influenced by a variety of factors, including the pathogen, patient characteristics (age, comorbidities), vegetation size and surgical proficiency. Combined antifungal therapy and surgical intervention achieve superior outcomes in *Candida* and *Aspergillus*-associated BCNE. Antifungal monotherapy demonstrates limited efficacy for *Aspergillus* BCNE, succeeding in only 2 of 53 cases (4%), versus 17 treatment successes (32%) with adjunctive surgical intervention [54]. Similarly, *Candida* endocarditis patients receiving combination therapy exhibited significantly higher survival rates (58% vs 41% with monotherapy) in a cohort of 103 cases [127]. Surgical resection of infected vegetations remains critical in *Aspergillus* BCNE management, eliminating niduses for catastrophic embolic complications and mortality [49].

Despite the challenges of surgical intervention and the risk of post-operative complications, the evidence appears to favor early surgery for the management of IE. However, it is important to consider the individual patient factors, and to take a multidisciplinary approach for making decisions in these patients.

## 6. Conclusion

BCNE has long been a diagnostic challenge, with delays in diagnosis contributing to increased mortality. While the management of BCNE remains complex, advancements in diagnostic modalities and a growing body of observational and epidemiological research have increased our understanding of effective management strategies.

The evolving epidemiology of BCNE, shaped by advancements in diagnostic techniques and the rise of antimicrobial resistance, underscores the importance of timely and accurate pathogen identification. Traditional methods such as imaging, histopathology, and serology remain valuable, while newer technologies such as PCR, TMGS, and SMGS have greatly enhanced the rapid identification of pathogens, though concerns regarding sensitivity and specificity persist. As pathogens continue to evolve, so too do optimal empiric treatment regimens. The potential role of early surgical intervention is an area of ongoing investigation.

Despite these advances, critical questions about BCNE remain unanswered. Continued research is essential to develop more efficient and precise diagnostic methodologies and to improve outcomes for patients affected by this condition.

## Abbreviations

IE, Infective endocarditis; BCNE, Blood culture-negative infective endocarditis; BCPE, Blood culture-positive infective endocarditis; PCR, Polymerase Chain Reaction; TTE, Transthoracic echocardiography; TMGS, Targeted metagenomics sequencing; SMGS, Shotgun metagenomics sequencing; PVE, Prosthetic valve infective endocarditis; NGS, Next-Generation Sequencing; WB, Western Blot; ESC, European Society of Cardiology; AHA, American Heart Association.
